# Does bariatric surgery influence plasma levels of fetuin-A and leukocyte cell-derived chemotaxin-2 in patients with type 2 diabetes mellitus?

**DOI:** 10.7717/peerj.4884

**Published:** 2018-06-12

**Authors:** Hsien-Hao Huang, Chun Yeh, Jung-Chien Chen, Tzong-Hsi Lee, Shu-Chun Chen, Wei-Jei Lee, Chih-Yen Chen

**Affiliations:** 1 Department of Emergency Medicine, Taipei Veterans General Hospital, Taipei, Taiwan; 2 Institute of Emergency and Critical Medicine, National Yang-Ming University School of Medicine, Taipei, Taiwan; 3 Division of Gastroenterology, Department of Internal Medicine, Cheng-Hsin General Hospital, Taipei, Taiwan; 4 Department of Internal Medicine, National Defense Medical Center, Taipei, Taiwan; 5 Department of Surgery, Min-Sheng General Hospital, Taoyuan, Taiwan; 6 Division of Gastroenterology and Hepatology, Department of Internal Medicine, Far Eastern Memorial Hospital, New Taipei City, Taiwan; 7 Department of Nursing, Oriental Institute of Technology, New Taipei, Taiwan; 8 Department of Nursing, Min-Sheng General Hospital, Taoyuan, Taiwan; 9 Taiwan Society for Metabolic and Bariatric Surgery, Taoyuan, Taiwan; 10 Division of Gastroenterology and Hepatology, Department of Medicine, Taipei Veterans General Hospital, Taipei, Taiwan; 11 Bariatric and Metabolic Surgery Center, Taipei Veterans General Hospital, Taipei, Taiwan; 12 Faculty of Medicine, National Yang-Ming University School of Medicine, Taipei, Taiwan; 13 Taiwan Association for the Study of Small Intestinal Diseases, Guishan, Taiwan; 14 Chinese Taipei Society for the Study of Obesity, Taipei, Taiwan

**Keywords:** Fetuin-A, Bariatric surgery, Type 2 diabetes mellitus, Leukocyte cell-derived chemotaxin-2, Obesity, Sleeve gastrectomy, Gastric bypass

## Abstract

**Background:**

Fetuin-A and leukocyte cell-derived chemotaxin-2 (LECT-2) are liver-derived proteins. Fetuin-A is an independent risk factor for type 2 diabetes (T2D) and obese patients with T2D have higher plasma fetuin-A levels than those without T2D. LECT-2 has positive correlation with the severity of both obesity and insulin resistance. The changes in plasma fetuin-A are not consistent after bariatric surgery and no studies have investigated the changes in LECT-2 on the obese patients with T2D after bariatric surgery.

**Methods:**

Overall, 18 patients undergoing gastric bypass (GB) and 16 patients undergoing sleeve gastrectomy (SG) were enrolled. The fasting plasma fetuin-A and LECT-2 levels were measured at baseline, one week, three months, and one year after surgery.

**Results:**

Both the GB and SG groups significantly decreased the body mass index (BMI), waist-to-hip ratio, a body shape index; the triglyceride, fasting blood sugar (FBS), hemoglobin A1c, C-peptide levels; and homeostatic model assessment (HOMA-IR) one year after surgery. The SG group showed a decreasing trend in plasma fetuin-A levels one year after SG surgery. There are no significant changes in LECT-2 one year after either GB or SG. Fetuin-A had a near significant negative relationship with insulin (*P* = 0.056) and HOMA-IR (*P* = 0.050) in the SG group. Changes in fetuin-A had a significant positive relationship with changes in BMI (*P* = 0.031) and waist-to-hip ratio (*P* = 0.031) in the GB group and had a near significant positive correlation with FBS (*P* = 0.051) in the SG group.

**Discussion:**

Neither GB nor SG modifies plasma levels of plasma fetuin-A or LECT-2 in T2D patients after surgery. The changes in plasma fetuin-A have a positive correlation with those of the BMI and waist-to-hip ratio 12 months after GB.

## Introduction

The incidence of obesity has tripled over the past 20 years (Hossain, Kawar & El Nahas, 2007). With this increasing prevalence of obesity, diabetes has also increased at the same rate as 90% of diabetes is attributed to excess weight (Hossain, Kawar & El Nahas, 2007). Obesity and type 2 diabetes (T2D), sometimes together called diabesity, have thus become a worldwide public health problem and financial burden (Ogurtsova et al., 2017; Chen et al., 2016; Ting et al., 2016). Metabolic liver disease has also been reported in 30 to 67% of obese patients (Luyckx et al., 1998; Blackburn & Mun, 2004). The European Association for the Study of the Liver, the European Association for the Study of Diabetes, the European Association for the Study of Obesity have established clinical practice guidelines for the management of non-alcoholic fatty liver disease (European Association for the Study of the Liver (EASL), European Association for the Study of Diabetes (EASD) & European Association for the Study of Obesity (EASO), 2016). Bariatric surgery is known to be the most effective treatment for obesity and can keep the promising long-term body weight loss in patients with morbid obesity (Higa et al., 2011; Sjöström et al., 2009; Sjöström et al., 2007; Syu, Inui & Chen, 2017). After 10 years, weight loss of 14 to 25% was maintained, depending on the type of bariatric surgery (Sjöström et al., 2007), and a mean excess weight loss of up to 57% was observed for gastric bypass (GB) surgery (Higa et al., 2011). A recent randomized trial has demonstrated that metabolic surgery, including gastric bypass and sleeve gastrectomy (SG), is as effective as surgical treatment in Asian patients who are non-morbidly obese (body mass index [BMI] < 35 kg/m^2^) with poorly-controlled T2D at one and two years after surgery (Lee et al., 2011a; Chen et al., 2013). Bariatric surgery (GB and SG) is known as the most effective and consistent method to improve metabolic syndrome, and has helped maintain body weight, glycemic control, and quality of life, when compared to medical therapy alone (Schauer et al., 2014). Moreover, both GB and SG had positive effects on metabolic liver disease (Mattar et al., 2005; Klein et al., 2006; Billeter et al., 2016).

Fetuin-A is a liver-derived protein and acts as an endogenous inhibitor of the insulin receptor tyrosine kinase (Mathews et al., 2000) in liver and skeletal muscle (Auberger et al., 1989). High levels of circulating fetuin-A are associated with insulin resistance (IR) (Mori et al., 2006; Stefan et al., 2006; Reinehr & Roth, 2008; Ali, Nassif & Abdelaziz, 2016), and a higher fetuin-A level is associated with a higher risk of diabetes (Aroner et al., 2017). Furthermore, fetuin-A is also an independent risk factor for T2D (Stefan et al., 2008). Obese patients with T2D have higher fetuin-A than non-T2D patients before bariatric surgery (Yang et al., 2015). Fetuin-A is positively correlated with metabolic liver disease and non-alcoholic fatty liver disease (Nascimbeni et al., 2018). Thus, fetuin-A levels are correlated with IR, obese T2D, and metabolic liver disease. After bariatric surgery, the changes in fetuin-A levels are not consistent and can either decrease (Brix et al., 2010; Jüllig et al., 2014; Yang et al., 2015) or remain unaffected (Kahraman et al., 2013; Verras et al., 2017). These discordant observations need further investigation.

Leukocyte cell-derived chemotaxin-2 (LECT-2) is a signaling molecule primarily expressed by hepatocytes (Yamagoe et al., 1997; Yamagoe, Mizuno & Suzuki, 1998) and regulates hepatic β-catenin (Ovejero et al., 2004) through Wnt/β-catenin pathways implicated in hepatic metabolism (Liu et al., 2011). Serum LECT-2 levels are increased in patients with obesity and fatty liver disease (Okumura et al., 2013; Yoo et al., 2017), suggesting that LECT-2 is a novel obesity-related protein (Okumura et al., 2013). Circulating LECT-2 levels positively correlate with the severity of both obesity and IR (Lan et al., 2014). However, there is few data available in the literature to explore changes in LECT-2 levels in obese patients with T2D after bariatric surgery.

In this study, our aim was to investigate the changes in fetuin-A and LECT-2 levels following bariatric surgery (GB and SG) and to correlate these alterations with other clinical parameters.

## Methods

### Patients and metabolic surgery

A hospital-based design was adopted in the present study. Patients with T2D who received either laparoscopic mini-gastric bypass (GB) or SG were enrolled into the present study. Briefly, patients were eligible for either surgical procedure according to the following-diagnostic and inclusive criteria: (1) T2D onset of more than 6 months with hemoglobin A1c (HbA1c) ≥8%, under the intensive medical care of an endocrinologist; (2) BMI  ≥25 kg/m^2^ and  ≤40 kg/m^2^; (3) willing to receive accessory therapy with diet control and exercise; (4) willing to undergo follow-up, and (5) willing to provide written informed consent.

Candidates were excluded if they (1) had cancer within 5 years of the study start; (2) were HIV-positive or had active pulmonary tuberculosis; (3) had cardiovascular diseases or cardiovascular instability within 6 months of study start; (4) had a history pulmonary embolism or uncontrolled coagulative diseases; (5) had serum creatinine levels >2 mg/dL, total bilirubin >2 mg/dL, prothrombin time prolonged >2 s, α-fetoprotein >20 ng/mL; (6) admitted to have chronic hepatitis B, C, liver cirrhosis, or inflammatory bowel diseases; (7) had acromegaly or receiving other organ transplantation; (8) underwent bariatric surgery, gastrointestinal surgery other than cholecystectomy, or had a prior abdominal septicemia; (9) had a history of alcohol or drug abuse, psychiatric diseases; or (10) presented uncooperative conditions.

Overall, 18 patients undergoing laparoscopic GB and an additional 16 patients undergoing laparoscopic SG were enrolled in this prospective, longitudinal study. The treatment decision was based on the predictors of diabetes remission after GB and SG, by using the Age, BMI, C-peptide, Duration of T2M (ABCD) score (Lee et al., 2013; Lee et al., 2015). SG was recommended for T2D patients with an ABCD score >4; thus, bariatric surgery with SG was recommended for T2D patients with younger age, high BMI, high C-peptide levels and short duration of T2D (Lee et al., 2015).

This study was conducted at the Department of Surgery of the Min-Sheng General Hospital and at the Taipei Veterans General Hospital, and was approved by the Ethics Committee of each hospital (approval number: MSIRB2015020).

### Surgical technique

GB was performed as described in our previous studies (Lee et al., 2011a; Lee et al., 2011b). In brief, we used a standard 5-port laparoscopic technique to create a long-sleeve gastric tube using the EndoGIA stapler (Tyco, US Surgical, Norwalk, CT, USA). The gastric tube was approximately 2.0 cm wide along with lesser curvature from the antrum to the angle of His. We also used an EndoGIA stapler to create a Billroth II type loop gastroenterostomy with the small bowel about 120 cm distal to the ligament of Treitz. There was no drain tube left in place. Using the mesh plug technique with bio-absorbable hemostatic gauze (Cellulostat, Horng Tzer Medical Instruments, Kaohsiung, Taiwan) (Chiu et al., 2006), we closed all the trocar wounds. GB is categorized as malabsorptive metabolic surgery that bypasses the foregut (especially the duodenum), and can achieve good T2D remission and weight loss (Lee et al., 2013). GB improves metabolic syndrome and metabolic hepatic disease (Mattar et al., 2005; Klein et al., 2006).

For SG, we used a laparoscopic stapler (EndoGIA; Coviden, Norvalk, CT, USA) with 60-cm cartridges (a 3.5-mm stapler height, blue load) to resect the greater curvature from the distal antrum (4 cm proximal to the pylorus) to the angle of His, including the complete fundus (Lee et al., 2011b). We left the remnant stomach tube, which was approximately 2 cm wide along the less curved side. The extended periumbilical trocar site was used for the extraction of the resected stomach portion. SG is volume restrictive metabolic surgery and also plays a role in T2D remission and weight loss (Lee et al., 2015). SG also has a good effect on metabolic hepatic disease (Billeter et al., 2016).

### Study protocol and anthropometric measurement

Two separate occasions of follow-up: at baseline (before surgery), as well as 12 months post-operatively, were performed for all participants. Routine laboratory tests, including serum total cholesterol (TC), triglyceride (TG), high-density lipoprotein cholesterol (HDL-C), low-density lipoprotein cholesterol (LDL-C), fasting blood sugar (FBS), HbA1c, and C-peptide, as well as anthropometric measurements were performed on each study day. The homeostatic model assessment (HOMA-IR) index, calculated according to the formula plasma glucose (mmol/L) × insulin (µU/mL) / 22.5, was measured and assessed IR (Lee et al., 2011a; Lee et al., 2011b). A body shape index (ABSI) was based on the waist circumference adjusted for height and weight (Krakauer & Krakauer, 2012).

In addition, four separate follow-up visits (at baseline before surgery, as well as at one week, three and 12 months post-operatively), were organized for all participants. Routine laboratory tests, including plasma fetuin-A and LECT-2 levels, were performed on each study day.

### Assays of plasma hepatokines levels

Venous blood samples were collected from the antecubital vein between 8 and 10 a.m. after an overnight fast before surgery, as well as at one week, three and 12 months after surgery. Blood samples were immediately transferred to a tube containing aprotinin (500 units/mL) (Lee et al., 2011a). After centrifugation at 300× g, plasma was separated, dispensed into polypropylene tubes in aliquots, and stored at −20 °C until analysis. Enzyme immuno-assays for plasma fetuin-A (R&D Systems, Minneapolis, MN, USA) and LECT-2 (Medical & Biological Laboratories CO., LTD., Nagoya, Japan), were carried out in a single batch run and in a blinded fashion, as described in our previous study (Lee et al., 2011a).

### Statistical analysis

All statistical analyses were performed using the Statistical Package for Social Sciences, version 12.01 (SPSS, Inc., Chicago, Illinois). Continuous variables were expressed as the mean ± standard deviation (SD). The chi-square test or Fisher’s exact test was used to compare categorical variables, while the Mann–Whitney *U* test was used to compare continuous variables. The Wilcoxon signed-rank test was used to compare baseline and post-operative variables. Friedman’s one-way analysis of variance followed by a *post hoc* test was used to analyze the differences among plasma levels of fetuin-A and LECT-2 before surgery, as well as one week, three and 12 months after surgery. Correlations between the two groups were examined using Spearman’s correlation analysis. A *P* value less than 0.05 was considered statistically significant.

## Results

### Treatment effect one year after bariatric surgery

**Figure 1:**
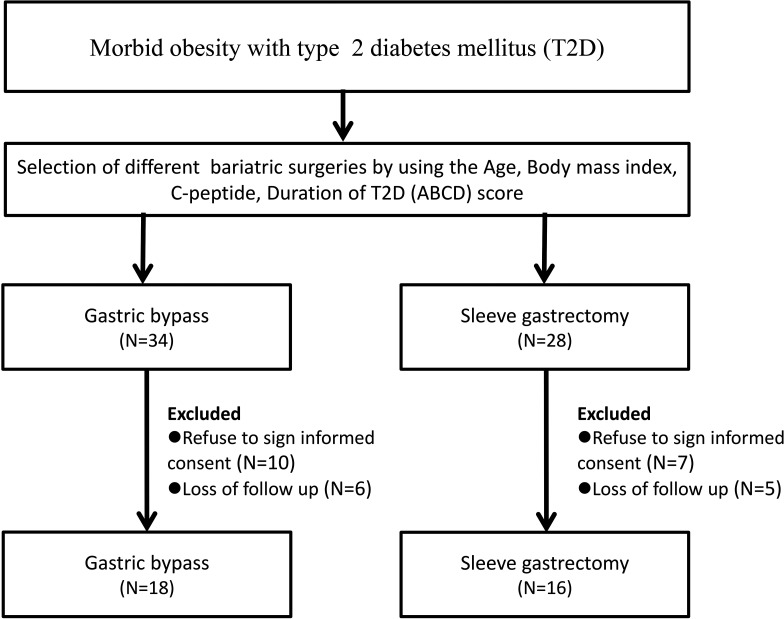
Flow chart of the patient selection process.

The flow chart of enrollment is shown in Fig. 1. In total, 18 patients (age: 42.8 ± 8.3 years, three males and 15 females) undergoing GB and 16 patients (age: 38.6 ± 9.2 years, 11 males and five females) undergoing SG were enrolled (Table 1). DM duration in the GB and SG groups was 4.0 ± 2.7 and 2.9 ± 3.0 years. Both the GB and SG groups significantly decreased the BMI, waist-to-hip ratio, ABSI, TG levels, FBS levels, HbA1c levels, C-peptide levels, and HOMA-IR one year after surgery (M12), as compared to before surgery measurements (M0).

Before surgery (M0), the SG group had significant higher a BMI (*P* < 0.01), lower ABSI (*P* < 0.05) and higher C-peptide levels (*P* < 0.01). One year after surgery (M12), the SG group had significant lower FBS (*P* < 0.05) and a higher C-peptide (*P* < 0.01) compared to the GB group.

**Table 1:** Characteristics of GB and SG patients before surgery (M0) and 1 year after surgery (M12).

	GB (*n* = 18)		SG (*n* = 16)	
	M0	M12	M0	M12
BMI (kg/m^2^)	30.700 ± 3.600	25.100 ± 2.200^###^	36.300 ± 5.600^**^	27.300 ± 4.600^###^
Waist-to-hip ratio	0.950 ± 0.048	0.869 ± 0.048^###^	0.959 ± 0.076	0.842 ± 0.076^###^
ABSI	0.082 ± 0.005	0.076 ± 0.004^###^	0.078 ± 0.005^*^	0.074 ± 0.006^#^
TC (mg/dL)	194.875 ± 45.716	166.875 ± 23.827^#^	193.286 ± 42.134	178.143 ± 29.501
TG (mg/dL)	202.000 ± 130.853	102.875 ± 52.262^###^	255.857 ± 155.109	97.286 ± 30.883^#^
HDL-C (mg/dL)	43.000 ± 7.510	45.375 ± 8.180	37.583 ± 7.204	49.000 ± 9.658^##^
LDL-C (mg/dL)	123.625 ± 32.114	107.125 ± 20.340	119.000 ± 33.059	111.929 ± 26.439
FBS (mg/dL)	165.412 ± 48.647	109.176 ± 33.787^###^	148.071 ± 59.285	90.500 ± 16.080^##^^*^
HbA1c (%)	9.141 ± 1.450	6.353 ± 0.873^###^	8.350 ± 1.794	5.864 ± 0.438^###^
Insulin (µU/mL)	18.158 ± 28.806	4.409 ± 3.327	13.155 ± 7.366	4.582 ± 2.679^###^
C-peptide (ng/mL)	2.562 ± 0.996	1.319 ± 0.560^###^	3.786 ± 1.521^**^	1.955 ± 0.496^#^^**^
HOMA-IR	6.647 ± 8.740	1.247 ± 1.125^#^	5.253 ± 4.247	0.974 ± 0.498^##^

**Notes.**

Abbreviations GB gastric bypass SG sleeve gastrectomy MO before surgery M12 months after surgery BMI body mass index ABSI a body shape index TC total cholesterol TG triglyceride HDL-C high-density lipoprotein cholesterol LDL-C low-density lipoprotein cholesterol FBS fasting blood sugar HOMA-IR homeostatic model assessment index insulin resistance

Asterisks indicates statistical difference between gastric bypass and sleeve gastrectomy groups:

* *P* < 0.05.

** *P* < 0.01.

£ indicates statistical difference between groups compared before and after surgery:

# *P* < 0.05.

## *P* < 0.01.

### *P* < 0.001.

### The changes in fetuin-A and LECT-2 levels one year after GB and SG surgery

**Figure 2:**
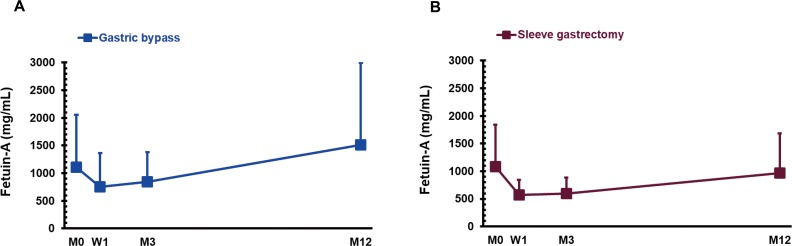
The serum concentration of fetuin-A in gastric bypass (A; *n* = 18) and sleeve gastrectomy (B; *n* = 16) patients before (M0), 1 week (W1), 3 months (M3), and 1 year (M12) after surgery.

**Figure 3:**
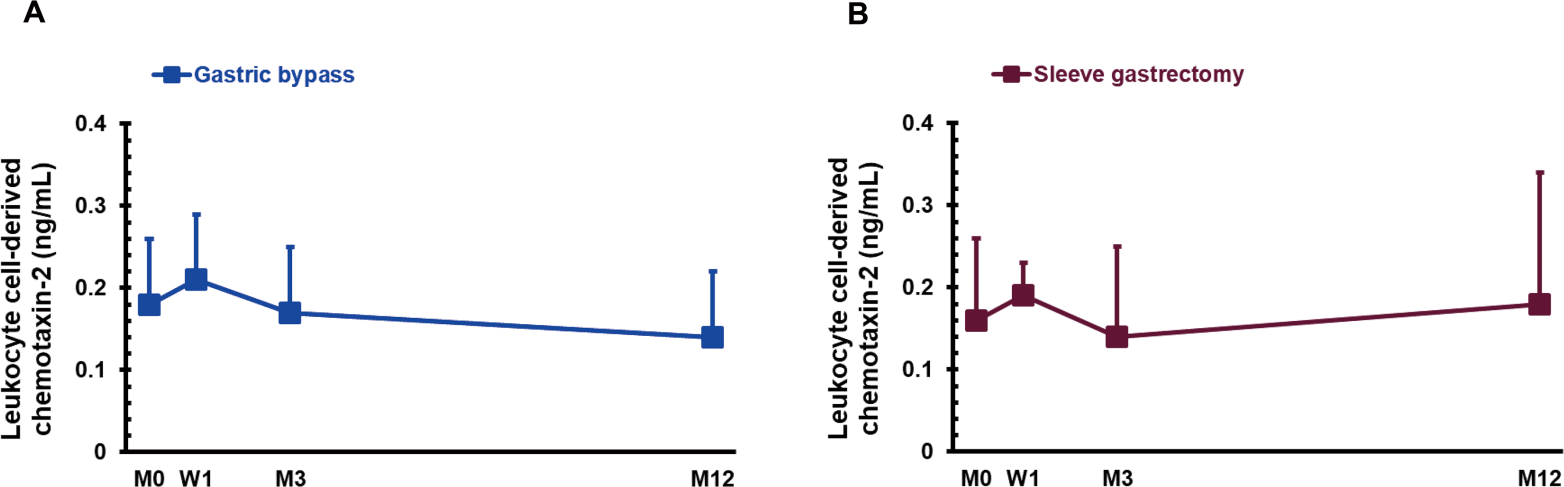
The serum concentration of leukocyte cell-derived chemotaxin-2 in gastric bypass (A; *n* = 18) and sleeve gastrectomy (B; *n* = 16) patients before (M0), 1 week (W1), 3 months (M3), and 1 year (M12) after surgery.

One year after SG surgery, GB group had no significant changes in fetuin-A (*P* > 0.05; Fig. 2A). Fetuin-A levels showed a decreasing trend (*P* = 0.072; Fig. 2B). There were no significant changes in LECT-2 at W1, M3 and M12 after GB or SG surgery (*P* > 0.05; Fig. 3).

We pooled these data (GB+SG = 34 cases), but did not detect any significant changes in fetuin-A (1,094.1 ± 852.0, 686.7 ± 508.5, 734.4 ± 459.4 vs. 1,254.7 ± 1,202.3 µg/mL) and LECT-2 (0.17 ± 0.09, 0.20 ± 0.06, 0.15 ± 0.09 vs. 0.16 ± 0.12 ng/mL) after bariatric surgery.

### Relationships between fetuin-A or LECT-2 and clinical parameters before surgery (M0) and one year after surgery (M12)

Before surgery (M0), fetuin-A showed no significant relationships with different parameters in the GB group (Table 2). Fetuin-A had a near significant negative relationship with insulin levels (*P* = 0.056) and the HOMA-IR index (*P* = 0.050) in the SG group. LECT-2 had no significant relationship with parameters in either the GB or SG group.

**Table 2:** Relationships between either fetuin-A or leukocyte cell-derived chemotaxin-2 levels and various parameters at baseline before surgery.

	GB (*n* = 18)				SG (*n* = 16)			
	Fetuin-A (µg/mL)		LECT-2 (ng/mL)		Fetuin-A (µg/mL)		LECT-2 (ng/mL)	
	rho	*P*	rho	*P*	rho	*P*	rho	*P*
BW (kg)	−0.107	0.662	0.014	0.954	−0.332	0.202	−0.0676	0.797
BMI (kg/m^2^)	0.027	0.908	−0.006	0.980	−0.335	0.198	−0.015	0.952
Waist-to-hip ratio	0.089	0.717	0.014	0.954	−0.081	0.754	0.056	0.831
ABSI	0.141	0.569	0.030	0.902	−0.032	0.900	−0.118	0.656
TC (mg/dL)	−0.265	0.281	−0.277	0.259	−0.471	0.073	0.357	0.185
TG (mg/dL)	−0.282	0.252	0.016	0.948	−0.343	0.186	0.205	0.436
HDL-C (mg/dL)	0.215	0.383	−0.348	0.153	0.100	0.705	0.239	0.366
LDL-C (mg/dL)	−0.136	0.580	−0.221	0.369	−0.074	0.780	0.347	0.182
FBS (mg/dL)	−0.145	0.558	0.239	0.334	−0.322	0.215	0.409	0.112
HbA1c (%)	0.056	0.818	0.096	0.699	−0.232	0.378	0.118	0.656
Insulin (µU/mL)	0.158	0.524	0.212	0.392	−0.500	0.056	0.200	0.465
C-peptide (ng/mL)	−0.267	0.278	0.146	0.558	0.221	0.403	0.185	0.483
HOMA-IR	0.129	0.603	0.290	0.238	−0.494	0.050	0.279	0.287

**Notes.**

Abbreviations GB gastric bypass SG sleeve gastrectomy BMI body mass index ABSI a body shape index TC total cholesterol TG triglyceride HDL-C high-density lipoprotein cholesterol LDL-C low-density lipoprotein cholesterol FBS fasting blood sugar HOMA-IR homeostatic model assessment index insulin resistance

One year after surgery (M12), fetuin-A had a significant, positive relationship with HbA1c (*P* = 0.033) in the GB group (Table 3) and showed only a trend for a negative relationship with TC (*P* = 0.087) in the SG group.

**Table 3:** Relationships between either fetuin-A or leukocyte cell-derived chemotaxin-2 levels and various parameters 12 months after surgery.

	GB (*n* = 18)				SG (*n* = 16)			
	Fetuin-A (µg/mL)		LECT-2 (ng/mL)		Fetuin-A (µg/mL)		LECT-2 (ng/mL)	
	rho	*P*	rho	*P*	rho	*P*	rho	*P*
BW (kg)	−0.256	0.312	−0.319	0.207	−0.020	0.94	−0.090	0.750
BMI (kg/m^2^)	−0.407	0.143	−0.271	0.340	−0.081	0.773	0.042	0.880
Waist-to-hip ratio	0.018	0.943	−0.422	0.099	0.358	0.221	−0.270	0.362
ABSI	0.385	0.167	−0.105	0.594	−0.104	0.723	−0.115	0.696
TC (mg/dL)	−0.152	0.563	−0.383	0.139	−0.470	0.087	−0.046	0.868
TG (mg/dL)	0.247	0.348	−0.395	0.127	−0.213	0.543	0.020	0.940
HDL-C (mg/dL)	−0.319	0.224	−0.140	0.594	−0.028	0.921	−0.118	0.682
LDL-C (mg/dL)	−0.009	0.969	−0.330	0.207	−0.420	0.129	0.020	0.940
FBS (mg/dL)	0.209	0.415	−0.424	0.087	−0.046	0.868	0.057	0.832
HbA1c (%)	0.517	0.033^*^	−0.319	0.207	0.222	0.435	−0.303	0.286
Insulin (µU/mL)	−0.368	0.156	−0.459	0.072	−0.300	0.304	0.045	0.878
C-peptide (ng/mL)	−0.403	0.118	−0.327	0.211	0.132	0.656	0.203	0.493
HOMA-IR	−0.265	0.314	−0.479	0.058	−0.368	0.206	0.005	0.978

**Notes.**

Abbreviations GB gastric bypass SG sleeve gastrectomy BMI body mass index ABSI a body shape index TC total cholesterol TG triglyceride HDL-C high-density lipoprotein cholesterol LDL-C low-density lipoprotein cholesterol FBS fasting blood sugar HOMA-IR homeostatic model assessment index insulin resistance

* *P* < 0.05.

### The relationships between change (Δ) in fetuin-A or LECT-2 and changes in various parameters one year after surgery (M12)

In Table 4, Δfetuin-A exhibited a significant, positive relationship with ΔBMI (*P* = 0.031) and Δwaist-to-hip ratio (*P* = 0.031) in the GB group, and had a near significant positive correlation with ΔFBS (*P* = 0.051) in the SG group. However, ΔLECT-2 did not reveal significant correlation with any parameters in either GB or SG groups.

**Table 4:** Relationships between changes in either fetuin-A or leukocyte cell-derived chemotaxin-2 levels and changes in various parameters 12 months after surgery.

	GB (*n* = 18)				SG (*n* = 16)			
	ΔFetuin-A (µg/mL)		ΔLECT-2 (ng/mL)		ΔFetuin-A (µg/mL)		ΔLECT-2 (ng/mL)	
	rho	*P*	rho	*P*	rho	*P*	rho	*P*
ΔBW (kg)	−0.320	0.203	0.0478	0.846	0.156	0.583	0.121	0.670
ΔBMI (kg/m^2^)	0.574	0.031^*^	−0.323	0.251	−0.244	0.390	0.002	0.988
ΔWaist-to-hip ratio	0.538	0.031^*^	−0.094	0.721	0.360	0.214	0.006	0.978
ΔABSI	0.187	0.511	−0.125	0.659	−0.115	0.696	0.071	0.806
ΔTC (mg/dL)	−0.347	0.182	−0.078	0.763	−0.427	0.121	0.015	0.952
ΔTG (mg/dL)	−0.113	0.664	0.137	0.601	0.341	0.244	0.203	0.493
ΔHDL-C (mg/dL)	0.065	0.805	−0.123	0.640	0.257	0.382	−0.512	0.070
ΔLDL-C (mg/dL)	−0.227	0.390	−0.052	0.839	−0.411	0.138	−0.279	0.324
ΔFBS (mg/dL)	0.429	0.083	0.223	0.382	0.525	0.051	−0.046	0.868
ΔHbA1c (%)	0.228	0.371	0.404	0.104	0.266	0.348	−0.266	0.348
ΔInsulin (µU/mL)	0.139	0.601	−0.209	0.429	0.033	0.906	0.159	0.591
ΔC-peptide (ng/mL)	0.079	0.763	−0.044	0.865	−0.374	0.199	−0.451	0.116
ΔHOMA-IR	0.388	0.133	−0.050	0.848	0.214	0.469	0.050	0.863

**Notes.**

Abbreviations GB gastric bypass SG sleeve gastrectomy MO before surgery M12 months after surgery BMI body mass index ABSI a body shape index TC total cholesterol TG triglyceride HDL-C high-density lipoprotein cholesterol LDL-C low-density lipoprotein cholesterol FBS fasting blood sugar HOMA-IR homeostatic model assessment index insulin resistance

* *P* < 0.05.

## Discussion

Our current study demonstrated that the BMI, waist-to-hip ratio, ABSI, TG levels, FBS levels, HbA1c levels, C-peptide levels, and HOMA-IR index were significantly decreased one year after either GB or SG, supporting the important role of bariatric surgery in maintaining long-term weight loss and improving glycemic control (Chen et al., 2013; Schauer et al., 2014; Yeh et al., 2017). However, neither GB nor SG showed any temporal effects on plasma levels of fetuin-A and LECT-2 at the one-year follow-up. Only the GB group had a positive relationship between changes in fetuin-A levels, as well as those in the BMI and waist-to-hip ratio.

Significantly higher fetuin-A levels of up to 877 µg/mL have been reported in the morbidly obese (Ix et al., 2008; Brix et al., 2010). In our study, the levels of fetuin-A were comparable with those of previous studies (Ix et al., 2008; Brix et al., 2010), supporting the concept that obesity is seemly one of the major factors for increased fetuin-A. Diabetes is another important related factor for increased fetuin-A levels. Patients with newly diagnosed T2D and impaired glucose tolerance have been reported to have higher serum fetuin-A concentrations than normal subjects (Ou et al., 2011). T2D patients have been documented to have higher fetuin-A concentrations than controls (Stefan et al., 2008; Ou et al., 2011; Sun et al., 2013; Yang et al., 2015), and plasma fetuin-A is independently and positively associated with a higher risk of developing T2D (Ix et al., 2008; Stefan et al., 2008; Sun et al., 2013), even after adjustment for age (Stefan et al., 2008) or exclusion of non-alcohol fatty liver disease (Ou et al., 2011).

**Table 5:** Summary of the temporal effects on changes in fetuin-A after bariatric surgery.

Morbidly obese (first & correspondence author)	Type of surgery	Number	BMI	T2D (number)	NAFLD (number)	Fetuin-A (length of follow-up)
Kahraman & Canbay et al., 2013 Clinical Science.	GB	108	53.3		Yes (108)	Increased (4M), Decreased (6M)
Verras & Kiortsis et al., 2017 Hormones	SG	20	42.5			Decreased (6M)
Yang & Lee et al., 2015 Obesity Surgery	GB SG	108 22	41.9 43.0	Yes (32) Yes (11)		Decreased (12M) Decreased (12M)
Brix & Schernthaner et al., 2010 The Journal of Clinical Endocrinology and Metabolism	GB	75	45.9			Decreased (16M)
Jüllig & Murphy et al., 2014 PLOS ONE	GB SG	8 7	42.1 42.3	Yes (8) Yes (7)		Decreased (3D) Decreased (3D)
Our current data	GB SG	18 16	30.7 36.3	Yes (18) Yes (16)		No effect (7D, 3M, 12M) Trend of decrease (7D, 3M, 12M)

**Notes.**

Abbreviations GB gastric bypass SG sleeve gastrectomy BMI body mass index T2D Type 2 diabetes mellitus NAFLD non-alcohol fatty liver disease D days M months

The literature is limited regarding the study of fetuin-A levels after bariatric surgery. In the obese, the concentration of fetuin-A has been shown to increase at four months (Kahraman et al., 2013), remain unchanged at six months (Kahraman et al., 2013), decrease at 12 months (Yang et al., 2015), or decrease at 16 months (Brix et al., 2010) in the morbidly obese after GB (Table 5). On the other hand, fetuin-A has also been showed to be either unchanged at 6 months (Verras et al., 2017) or decreased at 12 months (Yang et al., 2015) in the morbidly obese after SG. These results regarding fetuin-A levels after either GB or SG are very inconsistent. Diabetes, age, Caucasian origin, higher serum triglyceride levels, visceral adiposity, and nonalcoholic fatty liver disease are reported known related factors and require more investigation to control for potential confounding factors in the future (Ix et al., 2008; Stefan et al., 2008; Ou et al., 2011; Song et al., 2011; Haukeland et al., 2012; Sun et al., 2013; Stefan et al., 2014).

To the best of our knowledge, this study is the first to investigate the detailed temporal effects of bariatric surgery on fetuin-A levels in the obese with T2D. Fetuin-A has ever been reported to show an early significant decrease in the morbidly obese (BMI about 42) with T2D, three days after GB and SG (Jüllig et al., 2014). In our study, lower BMI (36.5 and 30.7 in SG and GB, i.e., mainly non-morbidly obese) might be the factor responsible for the non-significant decrease in the fetuin-A curve after surgery.

Serum concentrations of fetuin-A have been reported to be positively correlated with BMI in diabetics (Stefan et al., 2014) and in the obese (Erdmann et al., 2012; Ismail et al., 2012). Our study is the first to show that changes in fetuin-A levels had a positive correlation with those in BMI after GB surgery. A causal association has previously been proposed regarding circulating levels of fetuin-A and BMI (Thakkinstian et al., 2014).

Furthermore, fetuin-A has also been reported to be positive correlated with waist circumference (Ismail et al., 2012), while changes in fetuin-A have also shown to be positively correlated with waist circumference (Reinehr & Roth, 2008). In addition, fetuin-A levels were previously demonstrated to be positively correlated with waist-to-hip ratio one year after GB and SG (Yang et al., 2015). Our study is the first to reveal that changes in fetuin-A levels were positively correlated with those of waist-to-hip ratio one year after GB surgery. The changes in fetuin-A suggest re-evaluation as a therapeutic marker in obese patients with T2D after GB surgery.

To date, there is no information available regarding the influences of bariatric surgery on LECT-2. Our study showed LECT-2 levels did not change in obese patients with T2D after bariatric surgery. The possible use of LECT-2 as a therapeutic marker requires further investigation.

Our study had some limitations. First, the sample size was small. Second, a type-2 statistical error was due to the selected study subjects. Third, results of liver function testing , sonography, and liver histology were not available for all patients.

In summary, neither GB nor SG modifies plasma levels of plasma fetuin-A and LECT-2 in T2D patients after surgery. The changes in plasma fetuin-A show a positive correlation with changes in BMI and waist-to-hip ratio 12 months after GB.

##  Supplemental Information

10.7717/peerj.4884/supp-1 Supplemental Information 1 Fetuin-A and leukocyte cell-derived chemotaxin-2

## Additional Information and Declarations

### Data Availability

The following information was supplied regarding data availability: The raw data are provided in a Supplemental File.
